# Hyaluronic acid modified MPEG-*b*-PAE block copolymer aqueous micelles for efficient ophthalmic drug delivery of hydrophobic genistein

**DOI:** 10.1080/10717544.2018.1474972

**Published:** 2018-05-30

**Authors:** Cong Li, Rui Chen, Mengzhen Xu, Jiyan Qiao, Liang Yan, Xin Dong Guo

**Affiliations:** a Beijing Laboratory of Biomedical Materials, College of Materials Science and Engineering, Beijing University of Chemical Technology, Beijing, China;; b CAS Key Laboratory for Biomedical Effects of Nanomaterials and Nanosafety, National Center for Nanoscience and Technology, Beijing, China;; c Laboratory of Molecular Toxicology, State Key Laboratory of Integrated Management of Pest Insects and Rodents, Institute of Zoology, Chinese Academy of Sciences, Beijing, China;; d CAS Key Laboratory for Biomedical Effects of Nanomaterials and Nanosafety, Institute of High Energy Physics, Chinese Academy of Sciences, Beijing, China

**Keywords:** Polymer micelles, genistein, ocular delivery, cornea penetration, neovascularization inhibition

## Abstract

The ophthalmic drug delivery is a challenge in the clinical treatment of ocular diseases. The traditional drug administration usually shows apparent limitations, such as the low bioavailability from the reason of low penetration of the cornea and the short survival time of drug in the eyes. To overcome these shortcomings, we propose an amphiphilic polymer micelle modified with hyaluronic acid (HA) for high efficient ophthalmic delivery of genistein, a widely used hydrophobic drug for treatment of ocular angiogenesis. The MPEG-*b*-PAE copolymer was synthesized by the Michael addition reaction, and the final drug carrier MPEG-*b*-PAE-*g*-HA was obtained by the process of esterification. Then, genistein was packaged in this drug carrier, getting the final micelles with size of about 84.5 nm. The cell viability tests showed that the micelles take no obvious cytotoxicity to the human cornea epithelium cells. The functionalities of drug slow release and cornea penetration ability were demonstrated in a series *ex vivo* experiments. Further, the vascular inhibition test illustrated that the micelles could significantly inhibit the angiogenesis of human umbilical vein endothelial cells. These results indicate that the constructed polymer has high feasibility to be used as drug carrier in the treatment of ocular diseases.

## Introduction

A lot of proliferative neovascularization diseases, especially happens at the posterior part of the eye, could lead to serious ocular problems even the blindness (Witmer et al., 2003), such as wet age-related macular degeneration, proliferative diabetic retinopathy, and retinopathy of prematurity (Jager et al., 2008; Chuang et al., 2018; Xu et al., 2018). The critical mechanism of these diseases is the aberrant formation of new blood vessels in the eyes. Further researches showed that retinal pigment epithelial cells take the responsibility to secrete overhigh angiogenesis factors (such as VEGF) and the hypoxemia environment in the eye leads to the upregulation of various angiogenic factors (Gao et al., 2001; Farnoodian et al., 2017; Ioanna et al., 2018). Therefore, it is of great significance to study the treatment of these ocular neovascular diseases. However, the increasing of drug penetration to cornea is a pivotal step for achieving effective drug deliver to the posterior part of the eye in the treatment of ocular diseases.

At present, the treatment of eye diseases is 90% by topical administration, such as eye drops, eye ointment, and eye gel (Lim et al., 2016; Upadhayay et al., 2016; Xu et al., 2016). The traditional eye drops take advantages of convenience and good acceptance of the patients. However, due to reflex lacrimal secretion or blink, the drug stay only 1 ∼ 3 min in front of the cornea. And only about less than 5% of these drugs are eventually entered into the eye tissue because of the anterior corneal factors such as the tear dynamics (Villanueva et al., 2016). In order to overcome this difficulty, frequent and high doses of medication are required, which will lead to side effects and poor patient tolerance (Luo et al., 2011; Li et al., 2012; Liu et al., 2012). These shortages greatly limit the therapeutic effect of drugs on eye diseases. Therefore, researchers have focused on the drug delivery system recently. The first attempt was implants and there are many experiments with implants (Kane et al., 2008; Yavuz et al., 2016; Bisht et al., 2017; Verhoeven et al., 2018). However, the use of implants have side effects, such as increased intraocular pressure and cataract formation (Bakri & Omar, 2012; Sangwan et al., 2015). Liposomes are the most effective systems for the study of ocular diseases treatment, but they are mostly taken by systemic or intravenous administration, which will also cause lots of side effects (Singh et al., 2009; Allen & Cullis, 2013; Lajunen et al., 2016; Moustafa et al., 2017). Another meaningful treatment strategy is nanoparticles. Nanoparticles encapsulated drugs have been widely studied in ophthalmic drug delivery (Kang-Mieler et al., 2014; Balzus et al., 2017; Sanchez-Lopez et al., 2018). For nanoparticles, there are many pivotal factors, such as the particle size should be between 30 nm and 200 nm, the stability of biomolecules, the biocompatibility and absorbability of materials and so on. When administrated into the vitreous body, these nanoparticles can extend the drug release and reduce the number of injections. However, traditional intravitreal administration can cause complications, including ocular inflammation and elevated intraocular pressure. Therefore, the eye drops released by the sclera are the only noninvasive route to transport the drug to the posterior segment of the eye (Rafie et al., 2010).

In this research, we developed an amphipathic block copolymer modified by hyaluronic acid (HA) for serving as hydrophobic drug deliver carrier in treatment of ocular diseases. Genistein was used as the targeting delivery drug in this research for the reason that it shows reliable function for inhibiting the activity of tyrosine protein kinase (PTK) and angiogenesis (Wang et al., 2005; Ibrahim et al., 2010). Our research shows that the novel designed genistein-loaded micelles have the ability to inhibit neovascularization of the human umbilical vein endothelial cells (HUVECs), with having the diverse potential applications for clinical drug delivery.

## Materials and methods

### Materials

Methyl ether poly(ethylene glycol) (MPEG, Mn = 4000) and 3-amino-1-propanol (AP) were purchased from TCI (Tokyo, Japan). HA (Mn = 6800) obtained from Bloomage Freda Biopharm (Shandong, China). 1,6-hexanediol diacrylate (HDD), 4-dimethylaminopyridine (DMAP), and N,N′-dicyclohexylcarbodiimide (DCC) were acquired from J&K Scientific LTD (Beijing, China). Genistein was purchased from Selleck.cn (Shanghai, China).

### Synthesis of MPEG acrylate

MPEG (2 mmol) was dissolved in anhydrous DCM at 10% solids concentration in a round-bottomed flask. Triethylamine (4 mmol) was added and the contents were cooled to 0 °C and stirred half an hour, followed by the dropwise addition of acryloyl chloride (3 mmol). Then, keep stirring for extra 2 h at 0 °C, and keep stirring for a further 24 h at room temperature. The contents were extracted with dilute HCl solution two times, and then precipitated with hexane three times at −20 °C. The precipitates were dried in the vacuum drying oven for 48 h to obtain poly (ethylene glycol) methyl ether acrylate (MPEG-A), and the product was identified by ^1^H NMR (nuclear magnetic resonance).

### Synthesis of MPEG-*b*-PAE block copolymer

MPEG-*b*-PAE block copolymers were synthesized via a Michael-type step polymerization using MPEG-A as the monoacrylate, HDD as the diacrylate, and AP as the diamine, respectively. MPEG-A (0.2 mmol) and HDD (1.8 mmol) were dissolved respectively in anhydrous chloroform and then were mixed in solanic bottle. The bottle was injected nitrogen for 30 min at the same time heated up to 70 °C and then sealed, followed by the dropwise addition of AP (2 mmol). The reaction was carried out for 5 days at 70 °C under nitrogen. The copolymers were acquired after precipitated in hexane three times at −20 °C and dried in the vacuum drying oven, and then identified by ^1^H NMR.

### Synthesis of MPEG-*b*-PAE-g-HA copolymer

**Scheme 1.**
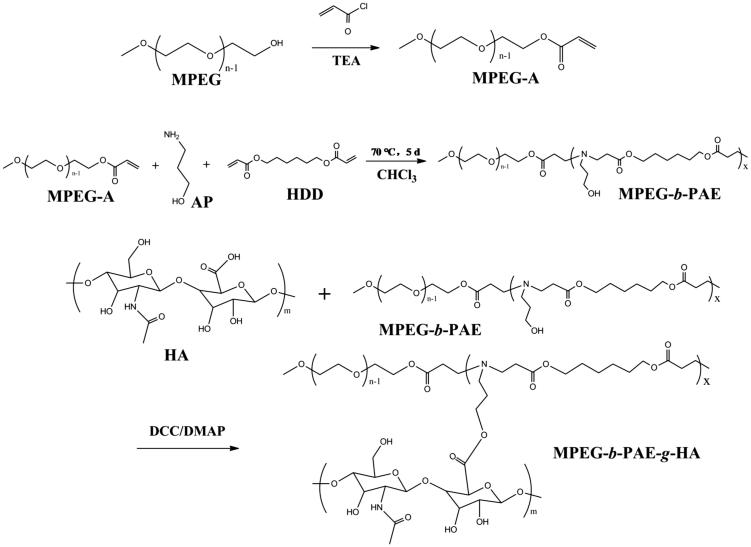
Schematic illustration for the synthetic route of MPEG-*b*-PAE-*g*-HA copolymer.

MPEG-*b*-PAE-*g*-HA copolymer was synthesized by esterification. HA (0.17 mmol), DCC (0.255 mmol), and DMAP (0.085 mmol) were dissolved respectively in anhydrous DMSO and then were mixed in a round-bottomed flask, followed by the dropwise addition of anhydrous DMSO solution of MPEG-*b*-PAE (0.085 mmol). The mixed solution was reacted for 24 h at room temperature. The final copolymers were acquired after precipitated in anhydrous ether three times and dried over 48 h and then identified by ^1^H NMR and GPC (gel permeation chromatography). The whole reaction route is shown in Scheme 1.

^1^H NMR spectra measurements were executed on a AVANCE III HD 400 spectrometer operating at 250 MHz, using deuterated chloroform (d-CDCl3) or deuterium oxide (D2O) as solvent and tetramethylsilane (TMS) as an internal standard. The temperature was 25 °C. The number average molecular weight (Mn) and polydispersity index (PDI, Mw/Mn) were determined by GPC adopting an Agilent 1200 series GPC system. The column system used HPLC grade THF as mobile phase with a flow rate of 1.0 mL/min at 30 °C.

### Preparation of genistein/MPEG-*b*-PAE-g-HA micelles

The spherical micelles of genistein/MPEG-*b*-PAE-*g*-HA were formed using the diafiltration method. In this experiment, 50 mg of MPEG-*b*-PAE-*g*-HA and 5-mg genistein were mixed and dissolved in 50 mL of DMSO with vigorous stirring for half an hour. The mixed solution was then dialyzed against 2 L of deionized water for 48 h at room temperature using a pre-swollen cellulose membrane bag (molecular weight cutoff, MWCO = 3500 Da). The deionized water was replaced every 2 h for the first 6 h and then every 5 h. After diafiltration, the solution was lyophilized using a freezing dryer for 2 days, and the drug-loaded micelles products were obtained in white powder. The preparation method of blank micelles was the same as above.

The size and zeta potential of micelles were determined by dynamic light scattering (DLS) with a Malvern Zetasizer Nano ZS. The detection concentration of micelles was 1 mg/mL. Morphologies of micelles were investigated by transmission electron microscopy (TEM, Tecnai G2 20 S-TWIN). For the TEM analysis, 50 μL of the micelle solution was dropped on 300 mesh copper TEM grids with a carbon film and then air-dried. GEN loading content (LC %) was determined by UV-vis spectrophotometer (UV-756MC) at 260 nm. A total of 1 mg GEN-loaded micelle powder was dissolved in 1 mL of DMSO. The concentration of GEN was calculated according to a standard curve of GEN/DMSO solution. The LC % was defined as the weight ratio of loaded drug to the drug-loaded micelles.

### *In vitro* release of GEN from MPEG-*b*-PAE-g-HA micelles

The release profiles of GEN from micelles were examined using a dialysis bag (MWCO =3500 Da) at 37 °C. The 5-mg GEN micelles were dissolved in the 500 μL PBS (pH 7.4, containing 2% Tween-80). The above solution was transferred in the dialysis bag and the bag was put into 5-mL PBS; the whole device was placed in a rocking bed at 110 rpm. At predetermined time intervals, 500 μL samples of the incubation medium were sampled and the whole medium was supplied with 500 μL fresh PBS. The drug concentration was analyzed using UV-vis spectrophotometry at 260 nm.

### Cell culture and *in vitro* cytotoxicity test

Human cornea epithelium cells (HCECs) and HUVECs were cultured in Dulbecco’s modified Eagle’s medium (DMEM, Gibco, Grand Island, NY, USA) supplemented with 10% fetal bovine serum (FBS, Gibco, Grand Island, NY, USA) at 37 °C, 5% CO_2_. *In vitro* cellular toxicity of genistein/MPEG-*b*-PAE-*g*-HA micelle was assessed by CCK-8 assay. Briefly, the cell density of cells was adjusted to 6 × 10^3^ cells/mL in DMEM, and then 100 μL aliquots of the cells were seeded at each well in a 96-well plate and incubated for 18 h. The cells were treated with 90 μL of the fresh medium and 10 μL of various concentrations of samples, and incubated at 37 °C in a humidified 5% CO_2_ incubator for 24 h, 48 h, and 72 h. At the same time, the blank control group was added to the 100 μL of the fresh medium without samples. Then 110 μL of the CCK solution was added to each well, and incubated in the incubator for 40 minutes at 37 °C. The absorbance was measured at 450 nm using a microplate reader. All data were represented as the average of three measurements.

### *In vitro* corneal penetration test

The corneal penetration experiment was carried out using a fresh rabbit cornea. All these tests compiled with the Guide for the Care and Use of Laboratory Animals, Institute of Laboratory Animal Resources. The rabbit was sacrificed by injection of air from the edge of the ear, and the cornea was carefully removed and immediately mounted on a vertical modified Franz Diffusion cell between the donor and receptor compartment (Li et al., 2012). The donor compartment was filled with 0.2 mL of either GEN eye drops or GEN-loaded micelle solutions with all containing the same quantity of genistein. The selected testing concentration of GEN-loaded micelle solution was 20 mg/mL. And the receptor compartment was filled with 5 mL of fresh PBS (pH 7.4, containing 2% Tween-80). The whole device was kept at 37 °C and stirred gently. At specific intervals, 0.5 mL of the release medium from the receptor compartment was collected for detection of GEN by UV-vis spectrophotometry at 260 nm, and an equal volume of freshly preheated PBS was added to the receptor compartment.

### Inhibition of neovascularization by micelles

*In vitro* angiogenesis of HUVECs was tested by 96-well plate method (Kureishi et al., 2000). First, 50 μL of the Matrigel (Discovery Labware Inc., USA) was added into the plate to taking an even spread at the bottom. Aseptic PBS was added around the hole of Matrigel to reduce evaporation, and then placed the 96-well plate into a humidified 5% CO_2_ incubator for 60 minutes to solidify the Matrigel. Then, the cell density of HUVECs was adjusted to 6 × 10^4^ cells per well, and drug-loaded micelle solution was prepared by the GEN at the concentration of 8 μg/mL. Finally, the cell suspension and the micellar solution were mixed evenly before adding to the pre-solidified Matrigel. The 96-well plate was placed in an incubator at 37 °C with 5% CO_2._ The cell suspension in the control group contained an equal volume of micellar dispersion instead of drug-loaded micellar solution. The neovascularization images were captured by an optical microscope (Olympus BX61W1 with Fluoview FV1000 software, Japan).

## Results and discussions

### Synthesis and characterization of the MPEG-*b*-PAE-g-HA copolymer

**Figure 1.**
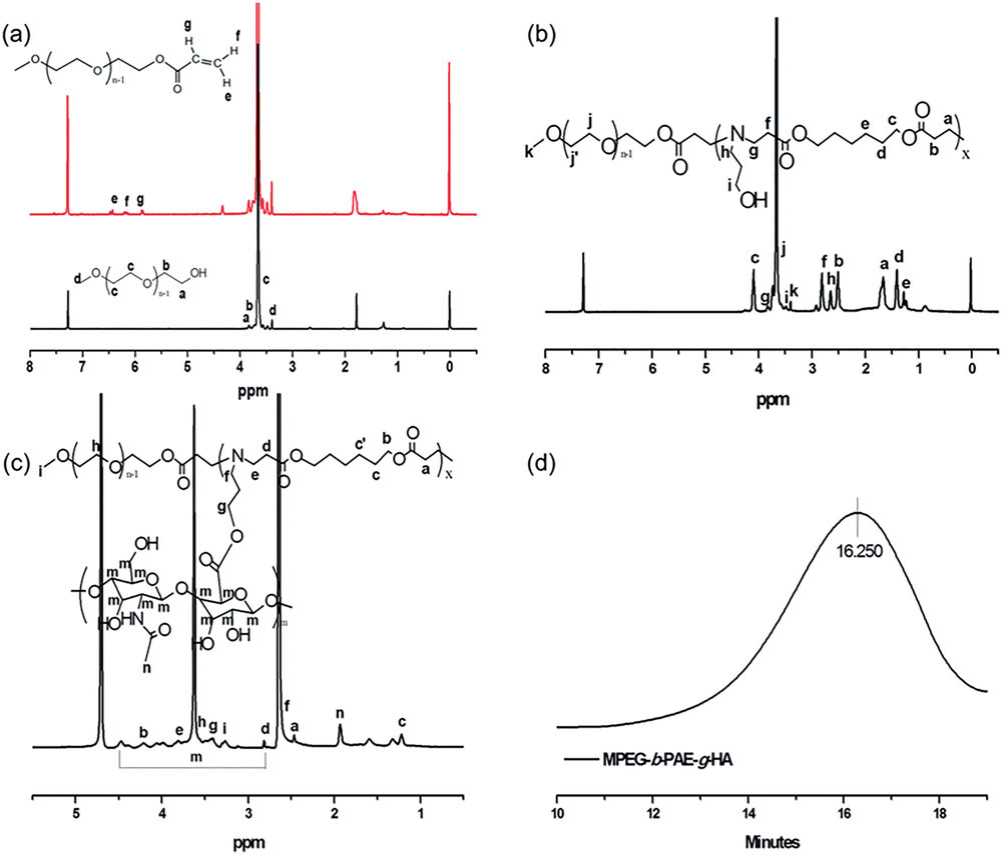
(a) ^1^H NMR spectra of MPEG (black line), MPEG-A (red line) in *d*-CDCl_3_. (b) ^1^H NMR spectra of MPEG-*b*-PAE in *d*-CDCl_3_. (c) ^1^H NMR spectrum of MPEG-*b*-PAE-*g*-HA in D_2_O. (d) GPC spectrum of MPEG-*b*-PAE-*g*-HA.

As shown in Scheme 1, the MPEG-*b*-PAE was obtained by the Michael-type step polymerization at 70 °C for 5 days, and the MPEG-*b*-PAE-*g*-HA was synthesized by esterification. Firstly, ^1 ^H NMR spectrum of MPEG-A was depicted in Figure 1(a), in which the up red line was MPEG-A. The characteristic peaks of the three protons on the terminal C=C double bond were 6.406–6.453 (e), 6.122–6.192 (f), and 5.829–5.859 (g) ppm, showing that the hydroxyl group at the end of MPEG became acrylate. The signal at 3.645 ppm was the characteristic peak of *–OCH_2_–CH_2_–* repeat unit in the MPEG chain segment. Figure 1(b) shows the ^1 ^H NMR spectrum of MPEG-*b*-PAE polymer. Compared with MPEG-A, three characteristic peaks on *–C=C–* double bonds disappeared, but there were characteristic peaks of PAE segment. The signals at h and i were ascribed to *–(CH_2_)_2_–CH_2_OH–* of the AP unit. The signals at g–f and a–e were due to *–(CH_2_)–COO–* and *–(CH_2_)_3_–COO–(CH_2_)_2_–* of the HDD units. The ^1 ^H NMR spectrum and GPC spectrum results of MPEG-*b*-PAE-*g*-HA copolymer are illustrated in Figure 1(c,d). From Figure 1(c), we can find that peaks of m were the characteristic peaks of HA, and the molecular weight of the final polymer was 11,738 as shown in Figure 1(d). Theses characterization results means that the designed MPEG-*b*-PAE-*g*-HA copolymer carrier was successfully synthesized.

### Characterization of the GEN-loaded micelles

**Figure 2.**
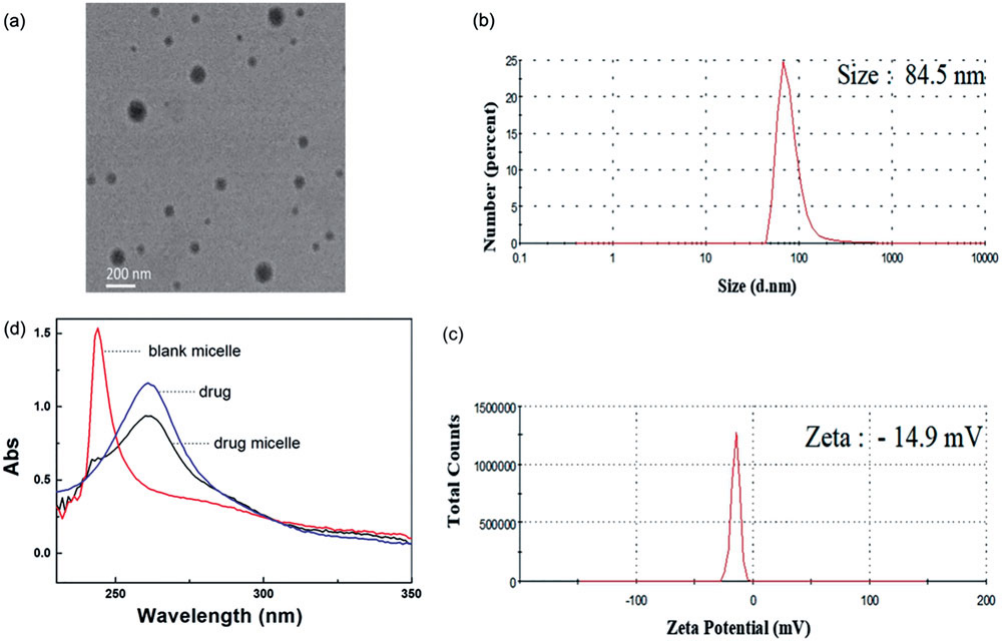
(a) TEM image of genistein/MPEG-*b*-PAE-*g*-HA micelles. (b) Particle size and (c) Zeta potential results of genistein/MPEG*-b*-PAE-*g*-HA micelle detected by dynamic light scattering. (d) Ultraviolet spectrophotometer of genistein (blue line), blank micelle (red line) and the drug-loaded micelle (black line).

The drug-loaded micelle was prepared by dialysis with involving the exchange of organic and aqueous phases. With the decrease of organic phase and the increase of water phase in the dialysis bag, the amphiphilic polymers gradually form core-shell micelles in the two-phase environment, and the micelles have hydrophobic core and hydrophilic shell. One of the functions of HA is to enhance the hydrophilicity of the micelles, so that the micelles take clear and transparent character in water. TEM results show that the polymer formed spherical micelles (Figure 2(a)).

**Figure 3.**
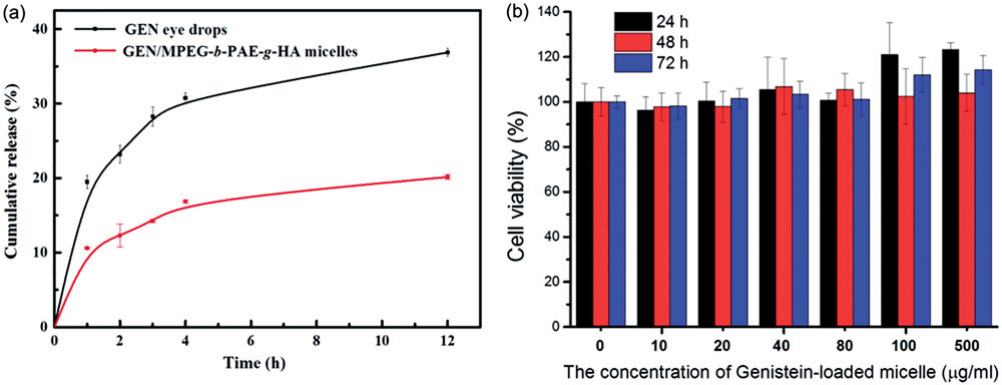
(a) *In vitro* drug release curves of drug-loading micelle and pure Genistein (*n* = 3). (b) *In vitro* cytotoxicity of the genistein/MPEG-*b*-PAE-*g*-HA micelles against HCECs after incubation for 24 h, 48 h, and 72 h (*n* = 5).

DLS was used to measure the hydrous particle size (Figure 2(b)) and zeta potential (Figure 3(c)). The average size of micelles was approximately 84.5 nm, and the average zeta potential was −14.9 mV. It has been shown that the transport capacity of the micelles has high relation to the sizes of the particles. The smaller of the particle size generally takes the higher corneal permeability for the micelles (Amrite & Kompella, 2005). Therefore, the size of micelles in this study is in line with the general requirement. The drug-loading capacity (LC %) of the drug-loaded micelles was detected by UV (Figure 2(d)). The absorption peak of the drug was at 260 nm, and the LC % was about 0.56% after the calculation according to the standard curve.

### *In vitro* drug release test

We used a dialysis bag method to evaluate release behavior on the drugs including the free genistein solution and the genistein-loaded micellar solution. Figure 3(a) shows the cumulative release percentage of released genistein in PBS (pH 7.4 containing 2% Tween-80, w/v) after incubation at 37 °C. Free genistein solution was diffused out faster from the dialysis bag than the genistein-loaded micelle aqueous solution. Within the first hour, the release percentage of free genistein was about 20%, while the drug-loaded micelle slightly higher than 10%, indicating that micelles could delay the drug release to some extent.

### *In vitro* cellular toxicity of GEN-loaded micelles

The HCECs were used as model cells for detecting the possible cytotoxicity of the genistein/MPEG-*b*-PAE-*g*-HA micelles. Figure 3(b) shows that the drug-loaded micelles have no obvious cytotoxicity against HCECs after incubation 24 h, 48 h, and 72 h with micelles concentrations from 10 to 500 μg/mL. Further test showed that the concentration of drugs contained in the micelle is indeed much lower than the LD50 of genistein (>15 μg/mL, Supplementary Figure S1). Therefore, the genistein/MPEG-*b*-PAE-*g*-HA micelles can be considered as a nontoxic carrier for ocular drug delivery.

### *In vitro* corneal penetration test

**Figure 4.**
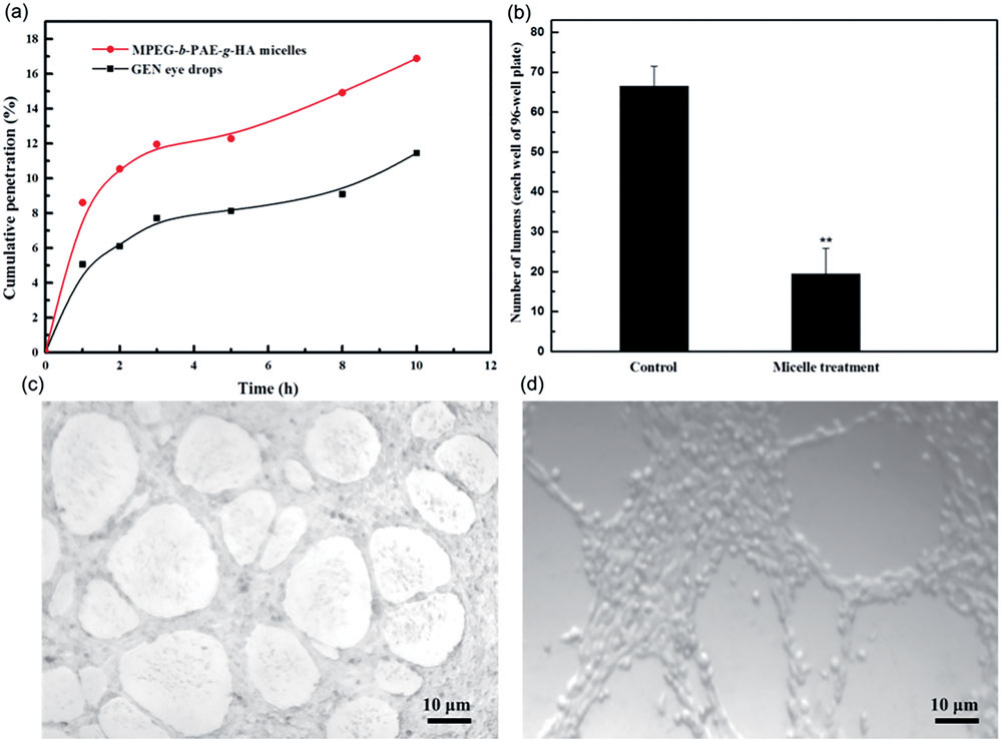
(a) *In vitro* corneal cumulative penetration profiles of genistein/MPEG-*b*-PAE-*g*-HA micelles and genistein eye drops. (b) The numbers of neovascular lumens of control groups and micelle treatment groups, *n* = 5. (c) Neovascular lumens formed by control HUVECs. (d) Vascular inhibition of genistein/MPEG-*b*-PAE-*g*-HA micelles on HUVECs.

A vertical modified Franz Diffusion cell was used to verify whether HA has the effect of improving corneal permeability. Figure 4(a) shows the cumulative penetration percentage of free genistein solution and genistein/MPEG-*b*-PAE-*g*-HA micelles with increasing time. In general, the genistein/MPEG-*b*-PAE-*g*-HA micelles showed higher permeability than the free genistein solution in PBS. Specifically, after 10 h, the cumulative penetration percentage of the genistein/MPEG-*b*-PAE-*g*-HA micelles and free genistein solution were 17% and 11%, respectively, and the permeability of drug-loaded micelle was approximately 1.5 times higher than the free genistein solution. The high permeability of genistein-loaded micelles for passing cornea may come from the reason of their amphiphilic character (Hennig & Goepferich, 2015). Furthermore, HA has mucosal adhesion ability, so that micelles modified with HA can improve the retention time of the system before the cornea, and then enhance the penetration on cornea. Therefore, the genistein/MPEG-*b*-PAE-*g*-HA micelles can improve the bioavailability of genistein after being used as eye drops.

### Inhibition of neovascularization by GEN-loaded micelles

A Matrigel tube formation assay was performed in the absence or presence of genistein/MPEG-*b*-PAE-*g*-HA micelles to study the inhibitory effect of drug-loaded micelles on the vascularization of HUVECs. Figure 4(b) shows that more capillary-like tubes were formed in the control group of HUVECs after culture for 24 h. Comparing with the control group (Figure 4(c)), the number of apparent tubes in micelle treatment group was significantly decreased and the lumen generally taking incomplete phenomenon (Figure 4(d)). It is worthy to note that the implement of this function did not pose significant cellular toxicity to HUVECs (Supplementary Figure S2). These results indicate that the genistein-loaded micelles could inhibit the angiogenesis of HUVECs. Therefore, the developed genistein/MPEG-*b*-PAE-*g*-HA micelles in this paper can be considered as a prominent option to treat ocular neovascularization.

## Conclusions

Polymer micelle has great development space as a drug carrier for ocular delivery. In this article, we investigated the possible functional applications of MPEG-*b*-PAE amphiphilic polymer micelles modified with HA, in which genistein was encapsulated in the micellar core for inhibiting angiogenesis. Generally the genistein is insoluble in water, so the hydrophobic nucleation of the polymer designed in this project can solubilize the genistein and form a transparent micellar solution. *In vitro* penetration testing results show that the corneal permeability of the micelles taking at least 1.5 times higher than pure drug solution. It may come from the reason that HA has the mucosal adhesion ability which can increase the adhesion of the carrier on the cornea, thereby increasing the probability of penetrating the cornea and increasing the utilization rate of the drug (Liu et al., 2008; Varghese et al., 2009; Oh et al., 2010). The results of *in vitro* vascular inhibition test of HUVEC cells disclosed that the micelles indeed took the function of inhibiting angiogenesis. These results illustrate that the designed micelles have the potential to improve the bioavailability of ocular drugs in the ocular diseases treatment.

## Supplementary Material

Supplemental Material

Supplemental Material
