# Supplementary material for: Hyaluronic acid modified MPEG-b-PAE block copolymer aqueous micelles for efficient ophthalmic drug delivery of hydrophobic genistein
Source: Drug Deliv. 2018 May 30;25(1):1258–65. doi: 10.1080/10717544.2018.1474972 (PMC6058726; doi:10.1080/10717544.2018.1474972)
Supplement: Supplemental Material [file IDRD_A_1474972_SM0152.docx]

**Supporting information**

**Hyaluronic acid modified MPEG-*b*-PAE block copolymer aqueous micelles for efficient ophthalmic drug delivery of hydrophobic genistein**

Cong Li^1^, Rui Chen^2, 3, *^, Mengzhen Xu^2^, Jiyan Qiao^2^, Liang Yan^4^, and Xin Dong Guo^1, *^

*^1^ Beijing Laboratory of Biomedical Materials, College of Materials Science and Engineering, Beijing University of Chemical Technology, Beijing 100029, China*

*^2^ CAS Key Laboratory for Biomedical Effects of Nanomaterials and Nanosafety, National Center for Nanoscience and Technology, Beijing 100190, China*

*^3^ Laboratory of Molecular Toxicology, State Key Laboratory of Integrated Management of Pest Insects and Rodents, Institute of Zoology, Chinese Academy of Sciences, Beijing 100101, China*

*^4^ CAS Key Laboratory for Biomedical Effects of Nanomaterials and Nanosafety, Institute of High Energy Physics, Chinese Academy of Sciences, Beijing 100049, China*

*** Corresponding authors:**

**Dr. Rui Chen, No. 11, Beiyitiao, Zhongguancun, Beijing 100190, China. Tel: +86-10-82545526. Fax: +86-10-62656765. E-mail: chenr@nanoctr.cn.**

**Prof. Xin Dong Guo, 15 Beisanhuandong Road, Chaoyang District, Beijing, China. Tel: +86-10-64451286. E-mail: xdguo@buct.edu.cn.**

**Figure S1.** Cellular toxicity of human cornea epithelium cells after incubation with Genistein for 24-h, 48-h and 72-h.

**Figure S2.** Cellular toxicity of HUVECs after incubation with genistein/MPEG-b-PAE-g-HA micelles for 24-h, 48-h and 72-h.
