# Supplementary material for: Hyaluronic acid modified MPEG-b-PAE block copolymer aqueous micelles for efficient ophthalmic drug delivery of hydrophobic genistein
Source: Drug Deliv. 2018 May 30;25(1):1258–65. doi: 10.1080/10717544.2018.1474972 (PMC6058726; doi:10.1080/10717544.2018.1474972)
Supplement: Supplemental Material [file IDRD_A_1474972_SM0151.doc]

**Responses to Reviewers’ Comments**

**Manuscript No.:** UDRD-2018-0203

**Title:** Hyaluronic acid modified MPEG-*b*-PAE block copolymer aqueous micelles for efficient ophthalmic drug delivery of hydrophobic Genistein

**Authors:** Cong Li, Rui Chen, Mengzhen Xu, Jiyan Qiao, Liang Yan, and XinDong Guo

**Reviewer #1**

In this work, the authors used the Michael addition reaction to synthesize an amphiphilic copolymers, which were modified by hyaluronic acid to form a MPEG-*b*-PAE-*g*-HA drug carrier for the delivery of genistein in the eye. Moreover, it was found that this system has inhibitory effect on neovascularization in the eye. This work is well organized and written. It can be accepted for publication after minor revisions.

**Comment**

Why MPEG and PAE are selected as the components of copolymer?

**Response**

Thanks to the reviewer’s comments. MPEG is a hydrophilic polymer with strong biocompatibility. PAE is a hydrophobic polymer with low toxicity and biodegradability. The two polymers can form a amphiphilic copolymer, which can be self-assembled into polymer micelles with hydrophobic core and hydrophilic shell in aqueous solution.

**Comment**

What is the effect of hyaluronic acid?

**Response**

Thanks to the reviewer for the constructive suggestion. Hyaluronic acid is one of the two main structural components of the vitreous body. HA has many functional properties, such as biocompatibility, biodegradability, non immunogenicity and non-toxic and so on. The most important thing is to extend the retention time of ophthalmic drugs by using the viscoelasticity and adhesion properties of HA in ocular drug delivery vectors.

**Comment**

Please modify the mailbox of the author of the Liang Yan.

**Response**

Thanks to the reviewer for the comments. According to the advice of the reviewer, the mailbox of the author of the Liang Yan has been modified.

**Comment**

Why the genistein is choosed for your experiment? Please introduction the most important function of genistein in the system.

**Response**

Thanks to the reviewer for the comment. One of the reasons is that genistein has the functions of inducing programmed cell death and inhibiting angiogenesis and so on. Another reason is that we want to study the inhibitory effect of drug loaded micelles on the neovascularization of the eyes. So we chose genistein as a model drug to study the properties of GEN/MPEG-*b*-PAE-*g*-HA micelles.

**Comment**

During the preparation of drug loaded micelles, 5 mg of genistein were selected for dialysis. Why not improve the quality of the drug to increase the drug loading?

**Response**

Thanks to the reviewer for the comments. Because when we increased the content of drugs, we found that precipitation occurs during dialysis. It may be because that when the ratio of drug content and polymer content is higher than 1:10, it exceeds the drug loading capacity of the polymer hydrophobic core. Thus after several experiments, 5 mg of genidtein was eventually selected for the preparation of drug loaded micelles.

**Comment**

Please explain that why the Tween-80 was added in PBS in corneal penetration and drug release experiments.

**Response**

Thanks to the reviewer for the comments. Genistein is an insoluble drug in water. If pure PBS was used as the medium, the drug would not be dissolved in the medium, and the content of the drug could not be detected. Tween-80 is a kind of surfactant, so adding Tween-80 can increase the solubility of drugs.

**Reviewer #2**

**Comment**

Why hyaluronic acid is used to modified the amphiphilic copolymer MPEG-*b*-PAE?

**Response**

Thanks to the reviewer for the careful review. The reason for chooseing hyaluronic acid is that HA has viscoelasticity and adhesion properties. MPEG-*b*-PAE modified by HA can enhance the retention time of the system on the cornea, and then increase the corneal permeability. The main purpose is to increase the bioavailability of the drug.

**Comment**

In cytotoxicity tests, human corneal epithelial cells are choosed to confirm the safety of materials, why human umbilical vein endothelial cells are also choosed?

**Response**

Thanks to the reviewer for the constructive suggestion. Our experiment is to study the ocular drug delivery system, so we need to evaluate whether the materials are non-cytotoxic to ocular cells. However, the aim of the designed system is to inhibit the neovascularization of the eyes, and HUVEC cells were used to produce new blood vessels, so it is also necessary to evaluate the cytotoxicity of the system to HUVEC cells.

**Comment**

The centigrade symbol in the submitted version can not be displayed properly. Please revise it before submitting again.

**Response**

Thanks to the reviewer for the constructive suggestion. According to the advice of the reviewer, the centigrade symbol format has been adjusted.

**Comment**

Why does the designed drug delivery system have the function of drug controlled release?

**Response**

Thanks to the reviewer for the comment. The polymeric micelles have a core-shell structure with hydrophobic core and hydrophilic shell. The hydrophobic core can encapsulate insoluble drugs. When the drug loaded micelles were dissolved in the aqueous solution and reached the diseased region, the drug is released gradually with the swelling of the polymer, thus reaching the purpose of drug controlled release.

**Comment**

Please change the letter b and g of MPEG-b-PAE-g-HA in the annotation of Figure 3 to italic and keep consistent with the text.

**Response**

Thanks to the reviewer for the comment. According to the advice of the reviewer, the letter b and g of MPEG-b-PAE-g-HA have been changed to italic in the annotation of Figure 3.

**Comment**

In Figure 4, you should provide the concentration of drug loading micelle treated with HUVECs.

**Response**

Thanks to the reviewer’s comments. In the part of the vascular inhibition experiment, the drug concentration in drug loaded micelles can be seen as 8 μg/ml.

**Comment**

In Figure 3a, the cumulative release of drug loading micelle was less than GEN eye drops in 12 hours. Dose that mean drug loading micelle have lower effectively than GEN eye drops in same period?

**Response**

Thanks to the reviewer for the comment. The cumulative release of the drug loaded micelles was less than that of the pure drug solution, which did not indicate that the efficiency of the drug loaded micelles is lower, but that the micelle structure has a controlled effect on the release of drugs. Drug loaded micelles can release drugs slowly as time gose on unlike pure drug solutions, which break up in a short time, resulting in sudden accumulation of drugs and low utilization.
